# The Intracellular Amastigote of Trypanosoma cruzi Maintains an Actively Beating Flagellum

**DOI:** 10.1128/mbio.03556-22

**Published:** 2023-02-22

**Authors:** Madalyn M. Won, Timothy Krüger, Markus Engstler, Barbara A. Burleigh

**Affiliations:** a Department of Immunology and Infectious Diseases, Harvard T.H. Chan School of Public Health, Boston, Massachusetts, USA; b Department of Cell and Developmental Biology, Biozentrum, University of Würzburg, Germany; Yale University School of Public Health

**Keywords:** kinetoplastida, *Trypanosoma cruzi*, cilia, flagella, host-pathogen interactions, intracellular parasites, mitochondria

## Abstract

Throughout its complex life cycle, the uniflagellate parasitic protist, Trypanosoma cruzi, adapts to different host environments by transitioning between elongated motile extracellular stages and a nonmotile intracellular amastigote stage that replicates in the cytoplasm of mammalian host cells. Intracellular T. cruzi amastigotes retain a short flagellum that extends beyond the opening of the flagellar pocket with access to the extracellular milieu. Contrary to the long-held view that the T. cruzi amastigote flagellum is inert, we report that this organelle is motile and displays quasiperiodic beating inside mammalian host cells. Kymograph analysis determined an average flagellar beat frequency of ~0.7 Hz for intracellular amastigotes and similar beat frequencies for extracellular amastigotes following their isolation from host cells. Inhibitor studies reveal that flagellar motility in T. cruzi amastigotes is critically dependent on parasite mitochondrial oxidative phosphorylation. These novel observations reveal that flagellar motility is an intrinsic property of T. cruzi amastigotes and suggest that this organelle may play an active role in the parasite infection process.

## OBSERVATION

**FIG 1:**
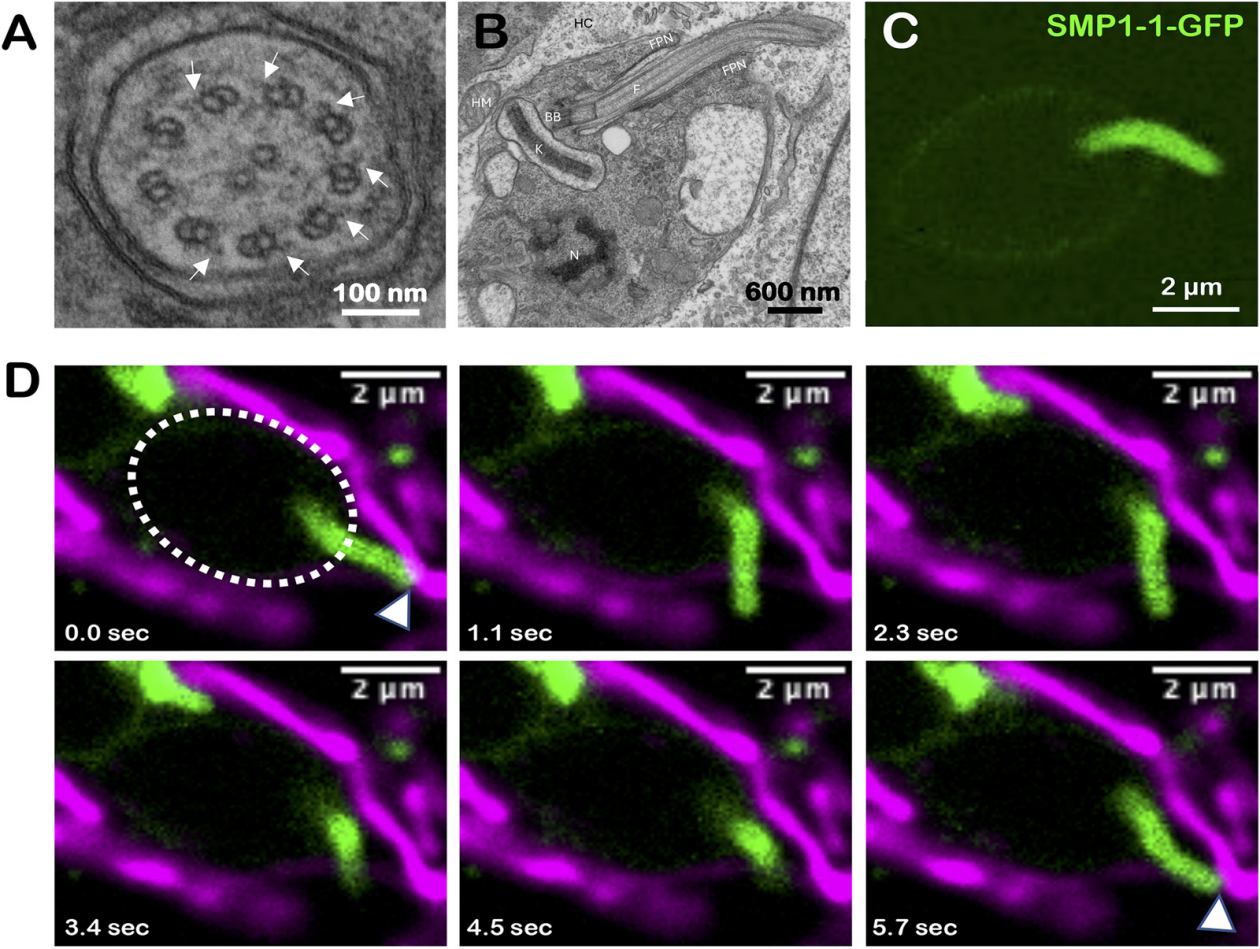
T. cruzi amastigotes have motile flagella. Transmission electron micrographs showing: (A) a representative cross-sectional view of a T. cruzi amastigote flagellum in the flagellar pocket neck region of the parasite, inside an NHDF, revealing the 9 + 2 axoneme and (B) a longitudinal section through an amastigote flagellum. Arrows in (A) indicate the visible dynein arms, abbreviations mark structures in (B) F, parasite flagellum; FPN, flagellar pocket neck; BB, basal body; N, parasite nucleus; K, kinetoplast; HC, host cell cytoplasm. (C) Fluorescence micrograph of a live SMP1-1-GFP-expressing T. cruzi amastigote. (D) Time-lapse images of an SMP1-1-GFP-expressing T. cruzi amastigote (*green*) are shown inside an NHDF expressing mitochondrially targeted mCherry (*pink*). The time each image was captured relative to the first frame in the series (arbitrarily set to 0.0 s) indicated at bottom of each frame. White outline of amastigote body added on first frame; white arrows indicate points of contact between the amastigote flagellum and host mitochondria.

The kinetoplastid protozoan parasite, Trypanosoma cruzi, is the causative agent of human Chagas disease and is associated with significant morbidity and mortality in Latin America (1). The ability of T. cruzi to establish intracellular residence in mammalian host cells, and to persist in diverse host tissues, are critical factors underlying disease progression. In mammals, intracellular infection is established by the nonreplicative, motile trypomastigote stage of T. cruzi (2). Once inside a host cell, trypomastigotes transition to the nonmotile “amastigote” stage, a morphologically and metabolically distinct T. cruzi life stage that proliferates in the cytoplasm of mammalian host cells (3–5). Accompanying the loss of motility in the amastigote is a dramatic shortening of the flagellum and loss of the paraflagellar rod (6), a lattice-like structure that runs parallel to the axoneme in motile trypanosomatid life stages (7). Intracellular T. cruzi amastigotes retain a short flagellum with a 9 + 2 axonemal structure (8) (Fig. 1A) that extends beyond the opening of the flagellar pocket with access to the extracellular milieu (Fig. 1B). Dynein arms, which typically serve as drivers of flagellar motility (9) in eukaryotes are visible on most doublets (Fig. 1A). Often described as a remnant and assumed to be inert (10), the T. cruzi amastigote flagellum has received little attention. The recent discovery of intimate contact between the flagellum of intracellular T. cruzi amastigotes and host mitochondria (11) has prompted speculation about possible functions of this vestigial amastigote flagellum (10, 11).

To gain insight into the spatiotemporal dynamics of the amastigote flagellum-host mitochondrial interaction, we generated transgenic T. cruzi parasites that stably express flagellar-targeted GFP (Fig. 1C; small myristoylated protein 1-1-GFP [SMP1-1-GFP], Text S1) (12). We first confirmed that the flagellar length and width were similar to that of the parental line (Fig. S1). The SMP1-1-GFP parasites were then used to establish intracellular infection in normal human dermal fibroblasts (NHDF; mitochondrial COX8-mCherry) and live fluorescence confocal imaging was performed at 48 hours postinfection (hpi), a time point at which intracellular amastigotes are in the exponential growth phase (3). Movement of the T. cruzi amastigote flagellum, relative to the parasite body and the host mitochondria, was immediately evident in these videos (Fig. 1D; Video S1), a time point at which intracellular amastigotes are in the exponential growth phase (3).

10.1128/mbio.03556-22.1 FIG S1 Automated length and width measurement of fluorescent flagella using Fiji, including the MorphoLibJ plugin. (A) Selected example image of focused flagellum showing length and width measurements (Fig. 1C; l = 3,03 μm, w = 0,97 μm). (B) Flagellum labelled by “Analyze Particles” after image A was binarized using “Auto threshold/Method Yen.” The dashed line shows the “Geodesic Diameter” as determined by the MorphoLibJ plugin and used for length measurement. (C) Geodesic distance map (MorphoLibJ) of the binarized image in B after using the plugin “Skeletonize” in order to determine the flagellum´s midline (black line). The highest value of the distance to the midline (color coded bright yellow) was used to calculate the width of the flagellum at the widest position (white line). (D) Flagellar length and (E) width (μm) distributions for parental Tulahuén amastigotes (untagged) fixed and stained with antibodies to flagellar calcium binding protein and SMP-1-GFP expressing amastigotes (*n* = 64; **, *P* < 0.01), both determined by the method presented in (A to C). Download FIG S1, TIF file, 3.9 MB. Copyright © 2023 Won et al. 2023 Won et al. https://creativecommons.org/licenses/by/4.0/ This content is distributed under the terms of the Creative Commons Attribution 4.0 International license.

Given that flagellar motility had never been described in the intracellular T. cruzi amastigote stage, we performed time-lapse imaging of the parental T. cruzi (Tulahuén) parasites by bright field microscopy (Video S2). These videos confirm that amastigote flagellar motility is independent of flagellar SMP1-1-GFP expression. We also demonstrate that flagellar beating occurs in intracellular T. cruzi amastigotes from genetically diverse backgrounds (Videos S3 to S5) and when resident in human (Fig. 1) or mouse cells (Video S6). Combined, these findings suggest that the previously unrecognized capacity for flagellar movement may be a universal property of intracellular T. cruzi amastigotes.

10.1128/mbio.03556-22.3 VIDEO S1 Live cell imaging of a T. cruzi Tulahuén strain amastigote expressing SMP1-1-GFP inside a normal human neonatal dermal fibroblast. Download Movie S1, MP4 file, 0.2 MB. Copyright © 2023 Won et al. 2023 Won et al. https://creativecommons.org/licenses/by/4.0/ This content is distributed under the terms of the Creative Commons Attribution 4.0 International license.

10.1128/mbio.03556-22.4 VIDEO S2 Live cell bright-field imaging of T. cruzi Tulahuén strain amastigotes inside a normal human neonatal dermal fibroblast. Arrows indicate visible flagella of amastigotes. Download Movie S2, MP4 file, 7.6 MB. Copyright © 2023 Won et al. 2023 Won et al. https://creativecommons.org/licenses/by/4.0/ This content is distributed under the terms of the Creative Commons Attribution 4.0 International license.

10.1128/mbio.03556-22.5 VIDEO S3 Live cell imaging of a T. cruzi CL Brener strain amastigote expressing SMP1-1-GFP inside a normal human neonatal dermal fibroblast. Download Movie S3, AVI file, 0.8 MB. Copyright © 2023 Won et al. 2023 Won et al. https://creativecommons.org/licenses/by/4.0/ This content is distributed under the terms of the Creative Commons Attribution 4.0 International license.

10.1128/mbio.03556-22.6 VIDEO S4 Live cell imaging of a T. cruzi Y strain amastigote expressing SMP1-1-GFP inside a normal human neonatal dermal fibroblast. Download Movie S4, AVI file, 0.8 MB. Copyright © 2023 Won et al. 2023 Won et al. https://creativecommons.org/licenses/by/4.0/ This content is distributed under the terms of the Creative Commons Attribution 4.0 International license.

10.1128/mbio.03556-22.7 VIDEO S5 Live cell imaging of a T. cruzi Brazil strain amastigote expressing SMP1-1-GFP inside a normal human neonatal dermal fibroblast. Download Movie S5, AVI file, 0.8 MB. Copyright © 2023 Won et al. 2023 Won et al. https://creativecommons.org/licenses/by/4.0/ This content is distributed under the terms of the Creative Commons Attribution 4.0 International license.

10.1128/mbio.03556-22.8 VIDEO S6 Live cell imaging of a T. cruzi Tulahuén strain amastigote expressing SMP1-1-GFP inside a mouse epithelial cell. Additional green signal above amastigote is another amastigote flagellum. Download Movie S6, MP4 file, 9.7 MB. Copyright © 2023 Won et al. 2023 Won et al. https://creativecommons.org/licenses/by/4.0/ This content is distributed under the terms of the Creative Commons Attribution 4.0 International license.

**FIG 2:**
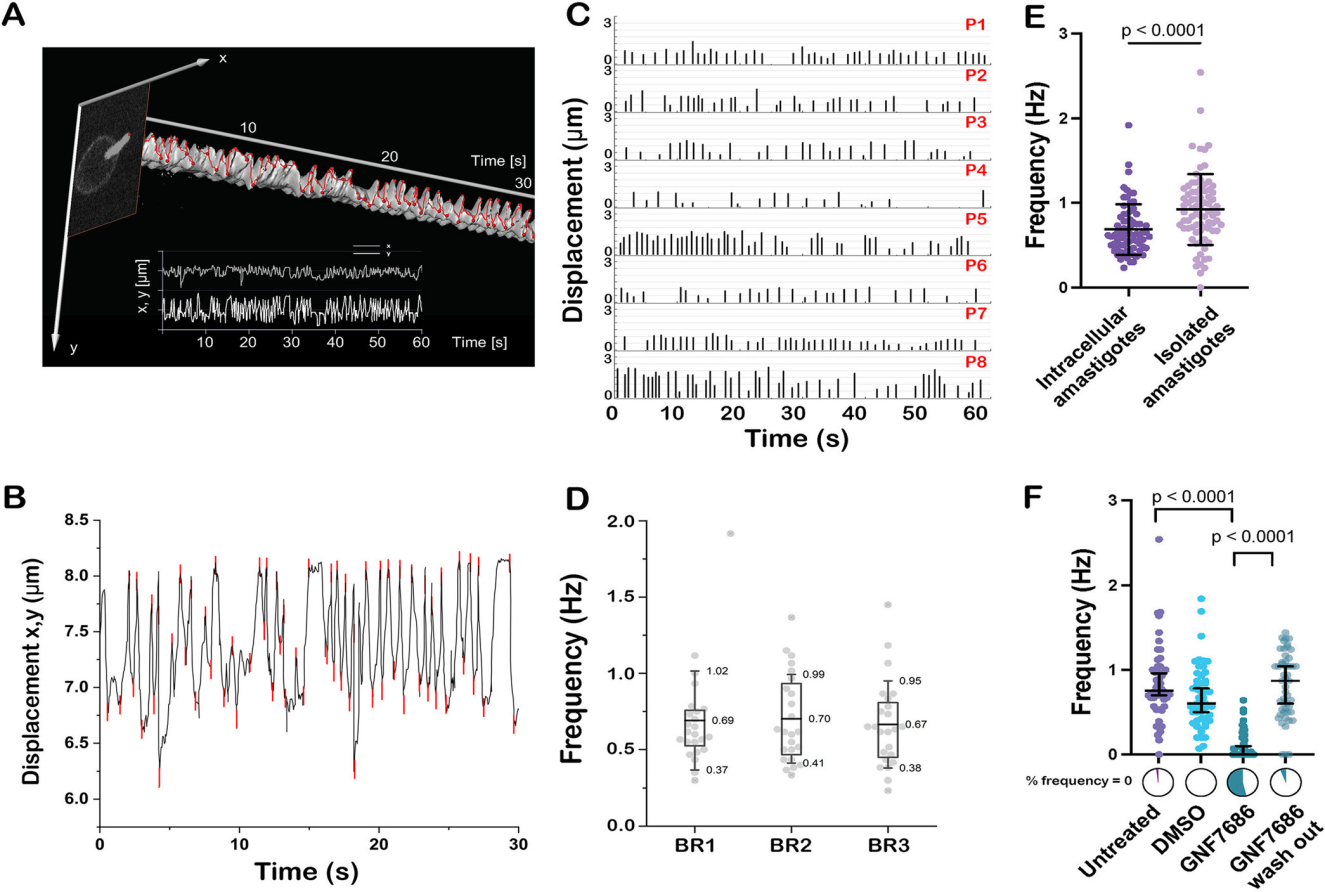
Quantifying amastigote flagellar beat. Analysis of fluorescence microscopy time-lapse series captured for host cell resident and isolated T. cruzi amastigotes reveals quasiperiodic flagellar beating. (A) An example of the trace amastigote flagellar tip tracking generated using Amira. (B) An example of peak analysis in origin with each peak shown in red. (C) Data from eight individual parasites (P1 to P8) showing the amplitude and temporal distribution of flagellar beats over 60 s following calculation of distances between amplitudes of tip location as specified by peak analysis shown in (B). (D) Aggregate data showing flagellar beat frequency for 24 individual parasites (gray dots) in each of 3 biological replicates (BR1 to 3). (E) Flagellar beat frequencies plotted for intracellular and isolated T. cruzi amastigotes (*n* ≥72), statistical analysis: Mann-Whitney test. (F) Amastigote flagellar beat frequencies observed under different treatment conditions: untreated control, DMSO (vehicle control), 10 μM GNF7686, and GNF7686 treatment followed by washout. Individual values for each parasite (circles) *n* ≥ 45, group shown with mean and standard deviation. Statistical analysis for (D and F), was a Kruskal Wallis test with Dunn’s multiple comparisons. Pie charts above labels show the percentage of parasite flagella in each group that did not move during (observation period) (frequency = 0) in color.

To determine the characteristics of flagellar motility in T. cruzi amastigotes, we analyzed a series of time lapse movies (*n* = 24) from each of three independent infection and imaging experiments. Amira software was employed to create kymographs as three-dimensional (3D) surface models of the fluorescence signal and to track the course of the flagellar tip for a duration of 60 s. This allowed the recording of absolute coordinates for the amplitude of tip movement (Fig. 2A). These data were then used to perform a peak analysis with OriginPro (Fig. 2B). The distance between successive peaks was calculated and plotted to generate precise and normalized displacement measurements for each flagellum independent of the orientation or position of the parasite cell body. The frequency, temporal distribution, and displacement are plotted for each flagellar beat over the observation period of 1 min for each parasite. Data obtained from eight individual parasites are shown (Fig. 2C, all parasites Fig. S2). The aggregated data for all parasites in each biological replicate is shown in Fig. 2D. Although the flagellar beat frequency is highly variable on an individual parasite level, the average flagellar beat frequency is 0.69 ± 0.30 Hz. Such quasiperiodic behavior can be expected in flagella shorter than 4 μm (13). Note that all measurements are necessarily confined to the imaging plane and thus quantify the apparent microscopic behavior routinely captured in 2D. We observed clear indications of three-dimensional, rotational movement of the flagella but presume that the current detailed description is sufficient for an initial analysis. Flagellar movement was consistently documented in intracellular T. cruzi amastigotes in the context of mammalian host cell infection. To uncouple amastigote flagellar motility from the potential influences of host cytoskeletal/organellar dynamics, SMP-1-1-GFP amastigotes were imaged following their release from mechanically disrupted host cells. Isolated T. cruzi amastigotes continue to beat their flagellum after separation from host cells, with beat frequencies like those measured for host cell resident amastigotes, albeit slightly faster (~0.2 Hz), likely from decreased viscosity of the medium versus the host cell cytoplasm (Fig. 2E). We also demonstrate that amastigote flagellar motility is severely impaired upon inhibition of amastigote mitochondrial respiration. Exposure of isolated amastigotes to GNF7686, a small molecule inhibitor that targets trypanosomatid *cytochrome b* (14) and blocks oxidative phosphorylation in amastigotes (15), resulted in severe impairment of flagellar movement with a significant increase in the proportion of parasites with no measurable flagellar movement (Fig. 2F). In line with the demonstration that GNF7686 lacks cytotoxicity toward T. cruzi amastigotes (3) flagellar beat resumed once the compound was washed out (Fig. 2F). Combined, these results demonstrate that flagellar beating is an intrinsic property of T. cruzi amastigotes and point to mitochondrial energy metabolism as a key driver of amastigote flagellar motility.

10.1128/mbio.03556-22.2 FIG S2 Individual intracellular amastigote flagellar beat profiles. Flagellar beat profiles showing the number, amplitude, and temporal distribution of beats per minute for all parasites in Fig. 2D. Download FIG S2, TIF file, 8.9 MB. Copyright © 2023 Won et al. 2023 Won et al. https://creativecommons.org/licenses/by/4.0/ This content is distributed under the terms of the Creative Commons Attribution 4.0 International license.

10.1128/mbio.03556-22.2 TEXT S1 Materials and Methods. Download TEXT S1, DOCX file, 0.03 MB. Copyright © 2023 Won et al. 2023 Won et al. https://creativecommons.org/licenses/by/4.0/ This content is distributed under the terms of the Creative Commons Attribution 4.0 International license.

In summary, this work has provided the first description of flagellar beating in the nonmotile intracellular amastigote stage of T. cruzi. Moreover, we now appreciate that the interaction between the T. cruzi amastigote flagellum and host mitochondria is dynamic, not (semi) stable as originally concluded from studies with fixed cells (11). While the biological role of amastigote flagellar motility has yet to be determined, the intracellular beating flagellum could alter the physical properties of the host cell cytoplasm, a shear-thinning fluid (16), to increase cytoplasmic fluidity and significantly influence diffusion dynamics locally (17). It could also facilitate metabolite sensing and/or uptake of nutrients via the flagellar pocket (18, 19) or the cytostome-cytopharynx complex (20). Future studies will be aimed at defining the role of amastigote flagellar beating in the context of intracellular infection, including the dynamics of the unique amastigote flagellum-host mitochondrial interaction (11).
