# Supplementary figures and images for: The Intracellular Amastigote of Trypanosoma cruzi Maintains an Actively Beating Flagellum
Source: mBio. 2023 Feb 22;14(2):e03556-22. doi: 10.1128/mbio.03556-22 (PMC10128032; doi:10.1128/mbio.03556-22)

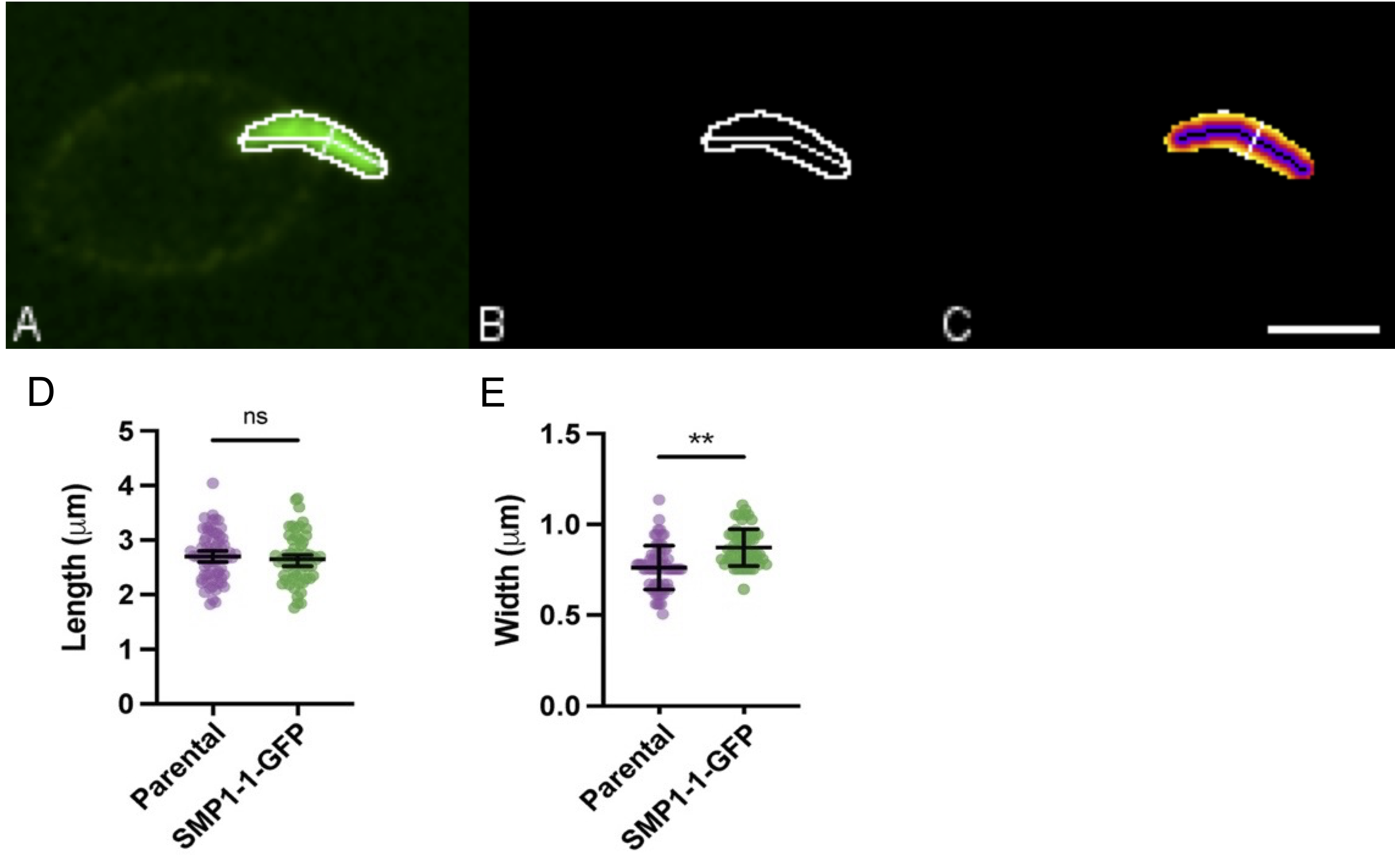

Supplement: FIG S1 [file mbio.03556-22-s0008.tif]
