# Supplementary material for: The Intracellular Amastigote of Trypanosoma cruzi Maintains an Actively Beating Flagellum
Source: mBio. 2023 Feb 22;14(2):e03556-22. doi: 10.1128/mbio.03556-22 (PMC10128032; doi:10.1128/mbio.03556-22)
Supplement: TEXT S1 [file mbio.03556-22-s0001.docx]

**Materials and Methods**

**Mammalian cell culture**

Mammalian cell lines were maintained in Dulbecco's modified Eagle medium (DMEM; HyClone, Logan, Utah, USA) supplemented with 10% heat-inactivated FBS (Gibco, Waltham, Massachusetts, USA), 25 mM glucose, 2 mM L-glutamine, and 100 U/mL penicillin-streptomycin (DMEM-10) at 37°C and 5% CO_2_. Normal human neonatal dermal fibroblasts (NHDF) were obtained from Lonza (Basel, Switzerland). Mouse embryonic fibroblasts (MEF) and monkey kidney epithelial cells (LLC-MK2) were purchased from the American Type Culture Collection (ATCC; Manassas, Virginia, USA). NHDF expressing mitochondrially-targeted mCherry were previously generated and maintained with G418 treatment prior to experiments ^1^.

***T. cruzi* cell culture**

*Trypanosoma cruzi* Tulahuén LacZ clone C4 was obtained from ATCC (PRA-330; Manassas, Virginia, USA). *T. cruzi* CL Brener, Y, and Brazil strains were obtained from Ricardo Gazzinelli (University of Massachusetts Medical School), Norma Andrews (University of Maryland) and Nisha Garg (University of Texas Medical Branch at Galveston), respectively. The axenic epimastigote stage of *T. cruzi* was propagated at 28°C in liver infusion tryptose (LIT) medium (4 g/L NaCl, 0.4 g/L KCl, 8 g/L Na_2_HPO_4_, 2 g/L dextrose, 3 g/L liver infusion broth, 5 g/L tryptose, with 25 mg/L hemin and 10% heat-inactivated FBS). To generate infectious metacyclic trypomastigotes, stationary phase *T. cruzi* epimastigotes were cultured in DMEM + 2% FBS (DMEM-2) for 5 days at 28°C. These metacyclic enriched cultures were washed in DMEM-2 and incubated for 2 days with confluent LLC-MK2 monolayers at 37°C, 5% CO_2_ to allow parasites to invade host cells. The mammalian stage trypomastigotes that emerged from infected LLC-MK2 cells were harvested from the cell culture supernatant by pelleting parasites at 2,060 x *g* for 10 minutes, then allowing trypomastigotes to swim up from the pellet for a minimum of 2 hr incubation at 37°C, 5% CO_2_ and collected. Purified trypomastigotes were then used to infect fresh LLc-MK2 cells in DMEM-2 to maintain the weekly mammalian-infective cycle or to establish intracellular infection prior to imaging.

**Generation of SMP1-1-GFP expressing *T. cruzi* lines.**

*T. cruzi* epimastigotes were transfected with a modified pTREX plasmid ^2^ containing the insert SMP1-1-eGFP followed by a P2A skip peptide, then a puromycin drug resistance cassette ^3^. The full-length SMP1-1 sequence (TcCLB.506563.200) was PCR amplified from genomic DNA isolated from *T. cruzi* Tulahuén strain parasites using the following primers: 5’-ATCGTAGAATTCATGGGCTGCGGTGCTTCTTCGAAACCCTCCAC-3'

5’-ATCGTAGCGGCCGCATTTTCTTTTCTTCTTCTTCCTTCGGGCGTTTGTTTTTTTCAGTGG

GGACGGC-3'. Prior to transfection, epimastigotes were pelleted at 2060 x g for 10 minutes, then resuspended in 100 μL of Tb BSF buffer ^4^. 4x10^7^ epimastigotes were loaded into a sterile 2 mm gap cuvette and transfected using an Amaxa Nucleofector II (Lonza, Basel, Switzerland; U-33 program). Parasites were immediately transferred to LIT medium for 24 hours before adding 10 μg/mL puromycin (Invivogen, San Diego, California, USA). After drug selection, SMP1-1-GFP expressing Tulahuén epimastigotes were cloned by limiting dilution in 96-well plates, and a single clone was selected for experimental use based on >90% SMP1-1-GFP expression. Uncloned populations of SMP1-1-GFP expressing Brazil, Y, or CL Brener strain parasites were used for imaging.

**Electron microscopy**

Procedure was completed as previously described ^1^. At 48 hpi, infected NHDF grown on Aclar (Ted Pella Inc., Redding, California, USA) filmed plastic coverslips were fixed with 1.25% formaldehyde, 2.5% glutaraldehyde, and 0.03% picric acid in 0.1 M sodium cacodylate buffer, pH 7.4 for 1 hour, and then washed 3 times in 0.1 M sodium cacodylate buffer (pH 7.4) prior to the post-fixation processing step of 1% osmium tetroxide/1.5% potassium ferrocyanide in distilled water 30 min on ice. Following three washes in distilled water, coverslips were incubated overnight with 1% aqueous uranyl acetate at 4 °C in the dark. Samples were rinsed in water and dehydrated in a graded ethanol series using the progressive lowering of temperature method. After a final dip in fresh 100% ethanol and then 100% propylene oxide, they were infiltrated with solutions 2:1, 1:2 of propylene oxide:epon araldite 30 min each, then 100% Epon araldite for 1 hour, then mounted for polymerization at 65 °C for 48 hr. Ultrathin sections (about 60 nm) were cut on a Reichert Ultracut-S microtome (Leica, Wetzlar, Germany), picked up on to copper grids stained with lead citrate, and examined in a TecnaiG2 Spirit BioTWIN (FEI Company, Hillsboro, Oregon, USA). Images were recorded with an AMT 2k CCD camera (Advanced Microscopy Techniques, Woburn, Massachusetts, USA).

**Indirect immunofluorescence microscopy and flagellar morphology quantification**

NHDF cells were seeded onto round glass coverslips (12 mm, #1.5; Electron Microscopy Sciences, Hatfield, Pennsylvania, USA) in 24-well plates at a density of 20,000 cells/well in DMEM-10 at 37^o^C, 5% CO_2_. Cells were infected the following day with WT Tulahuén trypomastigotes at a multiplicity of infection (MOI) of 3 suspended in DMEM-2, washed twice with PBS (Corning, Corning, New York, USA) 24 hours after infection and incubated for a further 24 hr in DMEM-2 at 37^o^C, 5% CO_2_. At 48 hours post-infection (hpi), the media was replaced with a 1% paraformaldehyde solution in PBS for a 10-minute incubation at 4°C. The following steps were carried out at room temperature, and each was preceded by three washes of the cells with PBS. Cells and parasites were permeabilized with 0.1% Triton-X 100 (v/v) (JT Baker, Phillipsburg, New Jersey, USA) for 10 minutes and a blocking solution of 3% (w/v) Bovine Serum Albumin (Sigma-Aldrich, St. Louis, Missouri, USA) in PBS for 1 hour. The primary antibody solution containing 1:1,500 rabbit α-FCaBP ^5^ in 1% BSA in PBS was added for 1 hour, followed by a 1:1000 α-Rabbit Alexa Flour 647 solution in 1% BSA in PBS for 1 hour. DAPI (0.2 μg/mL; Thermo Fisher Scientific, Waltham, Massachusetts, USA) in PBS was added for 5 minutes, and following washes, coverslips were placed onto slides with Prolong^®^ Diamond mounting medium (Thermo Fisher Scientific, Waltham, Massachusetts, USA). After setting for 24 hours, the cells were imaged with a 100x objective using a Yokogawa CSU-X1 spinning disk confocal system paired with a Nikon Ti-E inverted microscope and an iXon Ultra 888 EMCCD camera. Image processing, analysis, and display were completed using FIJI ^6^. Complete image analysis methods and an example are presented in **Fig S1**.

**Amastigote isolation**

Intracellular *T. cruzi* amastigotes were isolated from infected NHDF monolayers at 48 hpi as previously described ^7^. Briefly, infected cells were rinsed twice with PBS and dissociated from the culture flask using Accumax (Innovative Cell Technologies, San Diego, California, USA). Cell suspensions were washed twice in PBS before loading into gentleMACS M tubes (Miltenyi Biotec, Cologne, Germany) in a total volume of 2.5 mL. Infected host cells were lysed using the Protein_01.01 M tube protocol on the gentleMACS Dissociator (Miltenyi Biotec, Cologne, Germany) and the liberated *T. cruzi* amastigotes were pelleted at 2060 x g for 10 minutes and resuspended in imaging medium.

**Live cell confocal microscopy**

*Intracellular amastigotes*: 30,000 NHDF were seeded onto 35 mm glass bottom dishes (Matsunami Glass, Bellingham, Washington, USA) in DMEM-10 and allowed to attach for 24 hr at 37^o^C, 5% CO_2_. Cells were incubated with untagged or SMP1-1-GFP expressing *T. cruzi* trypomastigotes (MOI between 3 and 30) for 24 hr in DMEM-2 at 37^o^C, 5% CO_2_ to allow infection. The remaining extracellular parasites were removed by rinsing monolayers followed by further incubation in DMEM-2. At 48 hpi medium was aspirated and replaced with imaging medium: Fluorobrite™ DMEM (HyClone, Logan, Utah, USA) supplemented with 1.5% FBS, 2 mM glutamine, and ProLong™ Live Antifade Reagent (1:75; Invitrogen, Waltham, Massachusetts, USA). Dishes were imaged using a Yokogawa CSU-X1 spinning disk confocal system paired with a Nikon Ti-E inverted microscope equipped with a 37°C CO_2_ injectable environmental chamber and an iXon Ultra 888 EMCCD camera. All images were acquired with the 100x objective with a total imaging time between 30 seconds and 1 minute. Temporal resolution for intracellular amastigotes is 30 ms for the Tulahuén strain and 200 ms for the other *T. cruzi* strains in the supplemental videos. Dual channel imaging (**Supplemental Video 1**) has a temporal resolution of 1.08 s.

*Isolated amastigotes:* ~2 x 10^6^ freshly isolated intracellular *T. cruzi* amastigotes in 150 μL of pre-warmed imaging medium were placed directly onto the glass coverslip of a 35 mm glass bottom dish and allowed to settle at 37°C in a 5% CO_2_ incubator for 10 minutes. 75 μL of media was removed prior to placing the dish on the microscope for imaging. For GNF7876 treatment, 150 μL of isolated amastigotes were incubated with 10 µM GNF (Vitas-M Laboratory, Champaign, Illinois, USA) prepared from a 5 mM stock in DMSO for 10 min, then either directly transferred to the imaging well and allowed to settle for 10 min as above or for compound washout experiments, treated parasites were diluted 100-fold in PBS and centrifuged at 2060 x g for 10 minutes before resuspending in 150 µl imaging medium and proceeding as above. Temporal resolution for intracellular amastigotes is 80 ms.

**Quantification of flagellar beat**

*Intracellular amastigotes*: Kymographs of fluorescence microscopy time-lapse series were analyzed using Amira (2019.1, Thermo Fischer Scientific). Recordings of fluorescence-labeled flagella (SMP1-1-GFP) with a duration of 60 seconds (s) and a temporal resolution of 30 ms were used to create surface models using the timescale as the z-axis, thus creating 3d-kymographs of the fluorescence signal (e.g., **Fig. 2A**). Each surface model was rotated to a position in which the movement of the flagellum tip was clearly visible as an oscillating pattern on the surface model. The flagellar tip movement was then traced manually along the surface, creating a surface geodesic path (red track, **Fig. 2A**), yielding the coordinates of the tip in the plane of the original images for 60s time periods. The measurements of the x and y positions quantified the oscillations of the flagellum during the 60s recording. To generate a standardized and normalized dataset for the evaluation of beat frequency and amplitude, the x, y positions were exported and analyzed using the peak analysis tool of OriginPro (2021, OriginLab). The x and y values were converted into vector coordinates for peak identification in the positive and negative direction (e.g., **Fig. 2B**). The Euclidian distance between two successive peaks of opposite direction was then calculated, yielding the distance covered by the flagellum tip during each beat. This measurement of tip movement is independent of the cell orientation in the 2d image plane and thus normalizes the apparent amplitude of the flagellar beat (**Fig. 2C**). The tip movement between two peaks was significantly larger than movements of the tip caused by the diffusional displacement of the entire cell in the same time period, which could therefore simply be thresholded in the peak detection step. The average frequency [Hz] was calculated by dividing the number of measured beats by 60 [s].

*Isolated amastigotes*: The frequency of the extracellular amastigote flagellar beat was determined by counting the number of beats during visual inspection of the 60s time-lapse series. A beat was defined by an obvious bending of the axoneme, causing the flagellar tip to move a distance significantly further than any displacement of the cell body in the same time period, equivalent to the criteria used in peak analysis (see above). Statistical analysis was completed, as noted in figure legends using GraphPad Prism version 9.4.1 for Mac (GraphPad Software, San Diego, California, USA).

**References**

1. Lentini, G., Dos Santos Pacheco, N. & Burleigh, B. A. Targeting host mitochondria: A role for the *Trypanosoma cruzi* amastigote flagellum. *Cell. Microbiol.* **20**, (2018).

2. Dumoulin, P. C., Vollrath, J., Won, M. M., Wang, J. X. & Burleigh, B. A. Endogenous Sterol Synthesis Is Dispensable for *Trypanosoma cruzi* Epimastigote Growth but Not Stress Tolerance. *Front Microbiol* **13**, 937910 (2022).

3. Cormack, B. P., Valdivia, R. H. & Falkow, S. FACS-optimized mutants of the green fluorescent protein (GFP). *Gene* **173**, 33–38 (1996).

4. Schumann Burkard, G., Jutzi, P. & Roditi, I. Genome-wide RNAi screens in bloodstream form trypanosomes identify drug transporters. *Molecular and Biochemical Parasitology* **175**, 91–94 (2011).

5. Maric, D., Olson, C. L., Xu, X., Ames, J. B. & Engman, D. M. Calcium-dependent membrane association of a flagellar calcium sensor does not require calcium binding. *Mol Biochem Parasitol* **201**, 72–75 (2015).

6. Schindelin, J. *et al.* Fiji: an open-source platform for biological-image analysis. *Nat Methods* **9**, 676–682 (2012).

7. Dumoulin, P. C., Vollrath, J., Wang, J. X. & Burleigh, B. A. Glutamine metabolism modulates azole susceptibility in *Trypanosoma cruzi* amastigotes. *bioRxiv* 2020.06.19.161638 (2020) doi:10.1101/2020.06.19.161638.
